# Molecular epidemiology and mechanisms of tigecycline resistance in carbapenem‐resistant *Klebsiella pneumoniae* isolates

**DOI:** 10.1002/jcla.23506

**Published:** 2020-08-20

**Authors:** Yumi Park, Qute Choi, Gye Cheol Kwon, Sun Hoe Koo

**Affiliations:** ^1^ Department of Laboratory Medicine Chungnam National University Hospital Daejeon South Korea; ^2^ Department of Laboratory Medicine Konyang University College of Medicine Daejeon South Korea; ^3^ Department of Laboratory Medicine Konyang University Hospital Daejeon South Korea

**Keywords:** AcrAB‐TolC efflux pump, carbapenem‐resistant *Klebsiella pneumoniae*, tigecycline resistance

## Abstract

**Background:**

The emergence and transmission of tigecycline‐ and carbapenem‐resistant *Klebsiella pneumoniae* (TCRKP) have become a major concern to public health globally. Here, we investigated the molecular epidemiology and mechanisms of tigecycline resistance in carbapenem‐resistant *K pneumoniae* (CRKP) isolates.

**Methods:**

Forty‐five non‐duplicate CRKP isolates were collected from January 2017 to June 2019. We performed antimicrobial susceptibility tests, multilocus sequence typing (MLST), and pulsed‐field gel electrophoresis (PFGE). PCR and DNA sequencing were performed for the detection and mutation analysis of *acrR*, *oqxR*, *ramR*, *rpsJ*, *tet(A)*, and *tet(X)* genes, which are related to tigecycline resistance. The expression levels of efflux pump genes *acrB* and *oqxB* and their regulator genes *rarA*, *ramA*, *soxS*, and *marA* were assessed by quantitative real‐time PCR.

**Results:**

The resistance rate to tigecycline in CRKP isolates was 37.8% (17/45). *K pneumoniae* ST307 was a predominant clone type (70.6%, 12/17) among the TCRKP isolates. The expression levels of *acrB* (*P* < .001) and *marA* (*P* = .009) were significantly higher in the tigecycline‐resistant group than in the tigecycline‐intermediate and tigecycline‐susceptible groups. Increased expression of *acrB* was associated with *marA* expression (*r* = 0.59, *P* = .013).

**Conclusions:**

We found that the activated MarA‐induced overexpression of AcrAB efflux pump plays an important role in the emergence of tigecycline resistance in CRKP isolates.

## Graphical abstract

We introduce the mechanisms of tigecycline resistance in carbapenem‐resistant *K pneumoniae* (CRKP) isolates. The transcriptional activator MarA‐mediated overexpression of AcrAB efflux pump plays an important role in the emergence of tigecycline resistance in CRKP isolates.

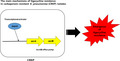

## 1 INTRODUCTION

As the emergence and dissemination of carbapenem‐resistant *Klebsiella pneumoniae* (CRKP) are being increasingly reported worldwide, multidrug‐resistant *K pneumoniae* is considered a serious global health threat.^1^, ^2^, ^3^, ^4^ Tigecycline, a glycylcycline antibiotic, has become one of the few available last resort antibiotics for the treatment of carbapenem‐resistant *Enterobacteriaceae* (CRE) infections such as CRKP.^5^, ^6^, ^7^, ^8^, ^9^ According to an international ESCMID cross‐sectional survey, tigecycline monotherapy is the most commonly used treatment for patients with intra‐abdominal infections and skin and soft‐tissue infections caused by CRE.^10^ However, CRKP isolates with tigecycline resistance have been reported.^11^, ^12^, ^13^ According to Taiwan's national surveillance study, the resistance rate of tigecycline (minimum inhibitory concentration, MIC > 2 mg/L) in carbapenem non‐susceptible *K pneumoniae* was reported to be 9%.^14^ In South Korea, the resistance rate of tigecycline (MIC > 2 mg/L) among carbapenemase‐producing *K pneumoniae* was found to be approximately 14.5%.^15^ Tigecycline resistance in CRKP has thus become a serious problem that can eventually lead to treatment failure.^11^, ^12^, ^13^

To date, there are several known mechanisms associated with resistance to tigecycline in *K pneumoniae* ^11^, ^16^, ^17^, ^18^, ^19^, ^20^, ^21^, ^22^, ^23^, ^24^, ^25^ The most common mechanism is the overproduction of non‐specific active resistance‐nodulation‐cell division (RND) efflux pumps such as AcrAB‐TolC^11^, ^19^ and OqxAB.^17^ Expression of the *acrAB* efflux pump genes is regulated by the global AraC‐family transcriptional activators such as RamA, MarA, and SoxS and the local TetR‐family transcriptional repressor AcrR.^16^, ^19^ The transcription of *ramA*, *marA*, and *soxS* is repressed by RamR, MarR, and SoxR, respectively.^13^, ^26^ RamA is also regulated by Lon protease.^27^, ^28^ As RamR directly represses the expression of *ramA*, loss‐of‐function mutations in *ramR* can cause the overexpression of *ramA*.^18^, ^21^ The expression of the *oqxAB* efflux pump genes is also regulated by the global activators RarA, MarA, and SoxS and the local repressor OqxR.^17^ Mutations in the *rpsJ* gene, which encodes the ribosomal protein S10, are associated with reduced tigecycline susceptibility.^24^ Moreover, mutations in *tet(A)*, which encodes one of the major facilitator superfamily (MFS) efflux pumps, and *tet(X)*, which encodes a tigecycline‐modifying enzyme, are associated with decreased tigecycline susceptibility.^20^, ^22^, ^23^, ^25^

The aim of this study was to investigate the phenotypic characteristics, molecular epidemiology, and mechanisms of tigecycline resistance in CRKP isolates from a tertiary care hospital in South Korea.

## 2 MATERIALS AND METHODS

### 2.1 Bacterial strains

A total of 3461 non‐duplicate *K pneumoniae* isolates were collected from Chungnam National University Hospital in South Korea from January 2017 to June 2019. The VITEK 2 ID‐GNB cards (bioMérieux SA, Marcy l’Étoile, France) were used for the identification of isolates. We retrospectively reviewed the clinical data for each isolate.

### 2.2 Antimicrobial susceptibility testing

The minimum inhibitory concentrations (MICs) of cefepime, cefotaxime, ceftazidime, gentamicin, amikacin, ciprofloxacin, trimethoprim/sulfamethoxazole, and chloramphenicol were determined using the E‐test (Biomerieux, Marcy l'Etoile, France) on Mueller‐Hinton (MH) agar (Difco Laboratories, Detroit, MI, USA) according to the Clinical and Laboratory Standards Institute (CLSI) guideline.^29^ Antimicrobial susceptibility to the carbapenems (ertapenem, imipenem, and meropenem) was determined by the agar dilution method, in accordance with the CLSI guidelines.^30^ The MICs for tigecycline (Sigma‐Aldrich, USA) were assessed by the broth microdilution method with MH broth (Difco Laboratories) in accordance with the European Committee on Antimicrobial Susceptibility Testing (2016 EUCAST) criteria (1.0 mg/L is susceptible, 2.0 mg/L is intermediate, and >2.0 mg/L is resistant).^31^ Both *Escherichia coli* ATCC 25922 and *Pseudomonas aeruginosa* ATCC 27853 were used as quality control strains for antimicrobial susceptibility testing. Tigecycline‐resistant and carbapenem‐resistant *K pneumoniae* (TCRKP) was defined if *K pneumoniae* was resistant to both at least one carbapenem [ertapenem (MIC ≥ 2.0 mg/L) and/or imipenem (MIC ≥ 4.0 mg/L) and/or meropenem (MIC ≥ 4.0 mg/L)] and tigecycline (MIC ≥ 4.0 mg/L).

### 2.3 Resistance gene detection

The following resistance genes were detected by PCR: (a) carbapenemase genes (*bla*_NDM_, *bla*_IMP_, *bla*_VIM_, *bla*_KPC_, *bla*_OXA‐48‐like_, and *bla*_GES_), (b) extended‐spectrum‐β‐lactamase (ESBL) genes (*bla*_CTX−M−1−like_, *bla*_CTX−M−9−like_, *bla*_TEM_, *and bla*_SHV_), (c) ampC β‐lactamase genes (*bla*_CIT_, *bla*_MOX_, *bla*_DHA_, *bla*_ACC_, *bla*_EBC_, *and bla*_FOX_), (d) quinolone resistance‐determinant genes (*qnrA*, *qnrB*, *qnrS*, *aac(6’)‐Ib‐cr*, and *qepA)*, and (e) aminoglycoside resistance‐determinant resistance genes (*armA*, *rmtA*, *rmtB*, and *rmtC*).^32^ The amplicons were determined by DNA sequencing. Primers used for PCR are shown in the Table S1.

### 2.4 Multilocus sequence typing (MLST) and pulsed‐field gel electrophoresis (PFGE)

MLST and PFGE were used to determine the genetic relatedness among the 45 CRKP isolates. PCR and sequencing for MLST were carried out for seven housekeeping genes (*gapA*, *infB*, *mdh*, *pgi*, *phoE*, *rpoB*, and *tonB)* for *K pneumoniae* and the sequences were compared in the MLST database, so that the allelic numbers and sequence types (STs) could be determined.^33^ The allelic profiles and STs were assigned using an online database (https://pubmlst.org/). For PFGE, bacterial DNA was cleaved with *Xba*I endonuclease (Roche, Penzberg, Germany), and the *Xba*I‐digested genomic DNA was subjected to PFGE using a CHEF‐DR^®^ III Variable Angle System (Bio‐Rad, USA).^34^ The PFGE patterns were compared using BioNumerics software (Applied Maths, Kortrijk, Belgium) with dice correlation for band matching at a 1.5% position tolerance and the unweighted pair group method with an arithmetic average (UPGMA). Clusters were defined as DNA patterns sharing >80% similarity.

### 2.5 Quantitative real‐time PCR (qRT‐PCR)

The expression levels of the efflux pump genes *acrB* and *oqxB* and their regulator genes *rarA*, *ramA*, *soxS*, and *marA* were assessed by qRT‐PCR. Total RNA of CRKP isolates was extracted with the MagListo^™^ 5M Cell Total RNA Extraction Kit (Bioneer, Daejeon, Korea) according to the manufacturer's instructions. Reverse transcription of RNA to cDNA was performed using AccuPower^®^ CycleScript RT Premix (Bioneer, Daejeon, Korea). Quantitative real‐time PCR performance using Solg^™^ 2X Real‐Time PCR Smart mix with EvaGreen^™^ 500 (SolGent Co., Ltd., Daejeon, Korea) was run on a Exicycler^™^ 96 Real‐time Quantitative Thermal Block (Bioneer, Daejeon, Korea). All experiments were performed in triplicate. The mRNA expression level of each gene was normalized based on an endogenous reference gene (*rrsE*). Relative expression of each gene was calculated based on tigecycline‐susceptible isolate CRKP 87 (tigecycline MIC 0.5 mg/L, expression = 1) as the control strain. The level of expression of each gene was calculated using 2^−ΔΔCt^ method. The primers used in the experiments are listed in the Table S2.

### 2.6 Detection and mutation analysis of the *acrR*, *oqxR*, *ramR*, *rpsJ*, *tet(A)*, and *tet(X)* genes and *pI* and*pII* promoter regions

We performed PCR to detect *acrR*, *oqxR*, *ramR*, *rpsJ*, *tet(A)*, and *tet(X)* genes and *pI* (upstream of the *romA* controlling *romA‐ramA* operon transcription) and *pII* (located in the open reading frame of *romA*) promoter regions, which are transcriptional start sites of the *ramA*.^11^, ^35^ The amplicons were sequenced. For mutation analysis, we compared each gene sequence with that of the wild‐type reference strains, *K pneumoniae* MGH78578 (GenBank accession number CP000647) in case of *acrR*, *oqxR*, *ramR*, and *rpsJ* genes and *pI* and *pII* promoter regions and *E coli* plasmid RP1 (GenBank accession number X00006) for the *tet(A)* gene. Primers used for PCR are shown in the Table S2.

### 2.7 Statistical analysis

All statistical analyses were performed using MedCalc statistical software 14.12.0 (MedCalc Software, Mariakerke, Belgium). Data are presented as the mean ± standard deviation (SD) unless otherwise stated. In groups with a non‐normal distribution, we evaluated the intergroup comparisons using either a Mann‐Whitney rank sum test or Kruskal‐Wallis test followed by Dunn's multiple comparison test. To assess the correlations between the expression levels of each gene, linear regressions were calculated. A *P* value < .05 was considered statistically significant.

## 3 RESULTS

### 3.1 Clinical characteristics of CRKP isolates

**Figure 1:**
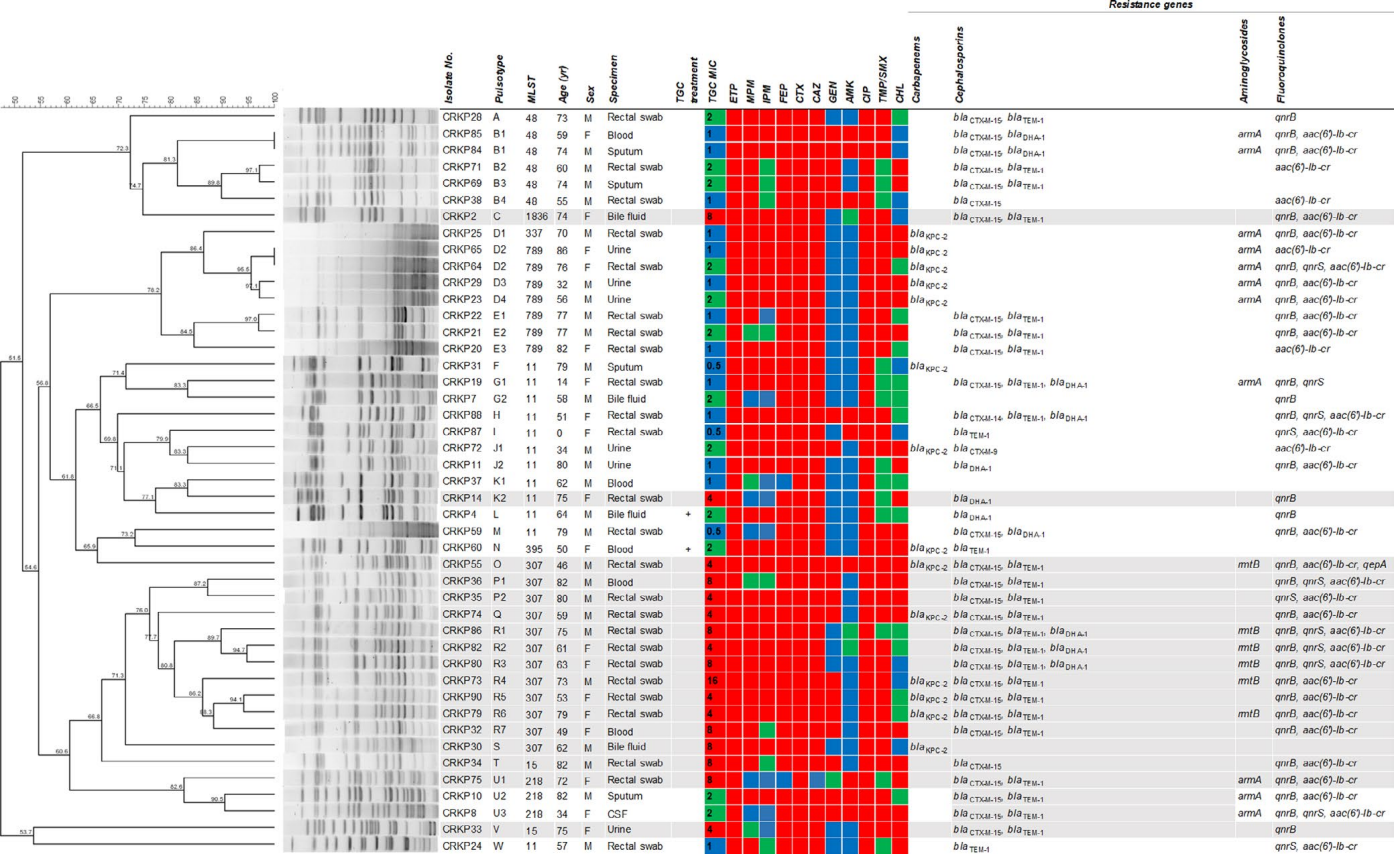
Dendrogram based on pulsed‐field gel electrophoresis patterns, multilocus sequence typing, characteristics, antibiotic susceptibility profile, and resistance gene profile of 45 carbapenem‐resistant *Klebsiella pneumoniae* isolates. The red, green, and blue squares indicate resistant, intermediate, and susceptible to each antibiotic, respectively. The gray background indicates 17 tigecycline‐ and carbapenem‐resistant *Klebsiella pneumoniae* (TCRKP) isolates. AMK, amikacin; CAZ, ceftazidime; CHL, chloramphenicol; CIP, ciprofloxacin; CTX, cefotaxime; ETP, ertapenem; FEP, cefepime; GEN, gentamicin; IPM, imipenem; MIC, minimum inhibitory concentration; MLST, multilocus sequence typing; MPM, meropenem; TGC, tigecycline; TMP/SMX, trimethoprim/sulfamethoxazole

Of the 3416 non‐duplicate *K pneumoniae* strains, CRKP accounted for 1.3% (45/3416). The isolates were obtained from various clinical specimens, including rectal swab (25/45, 55.6%), urine (6/45, 13.3%), blood (5/45, 11.1%), bile fluid (4/45, 8.9%), sputum (4/45, 8.9%), and cerebrospinal fluid (CSF) (1/45, 2.2%), from hospitalized patients aged 0 to 86, with a median age of 70 years (Figure 1). Two clinical *K pneumoniae* strains (CRKP4 and CRKP60) were isolated from patients who had previously been treated with tigecycline.

### 3.2 Antibiotic resistance profile and distribution of resistance genes in CRKP isolates

All CRKP isolates were resistant to ertapenem, cefotaxime, and ciprofloxacin. These isolates were also co‐resistant to ceftazidime (44/45, 97.8%), cefepime (43/45, 95.6%), meropenem (36/45, 80.0%), trimethoprim/sulfamethoxazole (32/45, 71.1%), imipenem (29/45, 64.4%), chloramphenicol (22/45, 48.9%), gentamicin (18/45, 40.0%), and amikacin (9/45, 20.0%) (Figure 1). Fourteen of 45 CRKP isolates (31.1%) harbored the carbapenemase genes *bla*_KPC‐2_. Except for two strains (CRKP7 and CRKP37), the remaining 29 carbapenemase non‐producing CRKP isolates had ESBL genes (27/29, 93.1%) such as *bla*_CTX‐M‐14_, *bla*_CTX‐M‐15_, and *bla*_TEM‐1_ and/or ampC lactamase such as *bla*_DHA‐1_ (14/29, 48.3%). Among the other resistance genes, the most commonly accompanied gene in CRKP was *aac(6')‐Ib‐cr* (34/45, 75.6%), followed by *qnrB* (32/45, 71.1%), *armA* (11/45, 24.4%), *qnrS* (11/45, 24.4%), *rmtB* (6/45, 13.3%), and *qepA* (1/45, 2.2%). The other resistant genes that were evaluated, such as carbapenemase genes (*bla*_NDM_, *bla*_IMP_, *bla*_VIM_, *bla*_OXA‐48‐like_, and *bla*_GES_), *bla*_SHV_, ampC β‐lactamase genes (*bla*_CIT_, *bla*_MOX_, *bla*_ACC_, *bla*_EBC_, and *bla*_FOX_), *qnrA*, *rmtA*, *and rmtC,* were not detected in the CRKP isolates. Among CRKP isolates, the prevalence of tigecycline non‐susceptible (MICs ≥ 2 mg/L) strains was 64.4% (29/45) and that of tigecycline‐resistant (MICs ≥ 4 mg/L) strains was 37.8% (17/45). Among the 17 TCRKP isolates, six (35.3%) harbored *bla*_KPC‐2_.

### 3.3 Molecular epidemiology based on MLST and PFGE

Nine distinct STs were observed among the 45 CRKP isolates as follows: ST11 (12/45, 26.7%), ST307 (12/45, 26.7%), ST789 (7/45, 15.6%), ST48 (6/45, 13.3%), ST218 (3/45, 6.7%), ST15 (2/45, 4.4%), ST337 (1/45, 2.2%), ST395 (1/45, 2.2%), and ST1836 (1/45, 2.2%) (Figure 1). For PFGE analysis, 43 different pulsotypes and 23 clonal groups were observed among the 45 CRKP isolates. Among 17 TCRKP isolates, five distinct STs, which were differentiated into 17 pulsotypes and 10 clonal groups, were observed as follows: ST307 (12/17, 70.6%, PFGE pulsotype O, P1‐P2, Q, R1‐R7, S), ST15 (2/17, 11.8%, PFGE pulsotype T, V), ST11 (1/17, 5.9%, PFGE pulsotype K2), ST1836 (1/17, 5.9%, PFGE pulsotype C), and ST218 (1/17, 5.9%, PFGE pulsotype U1).

### 3.4 Gene expression level analysis of efflux pumps *acrB* and *oqxB* and their regulators *rarA*, *ramA*, *soxS*, and *marA* in the tigecycline‐susceptible, intermediate, and resistant CRKP groups

**Figure 2:**
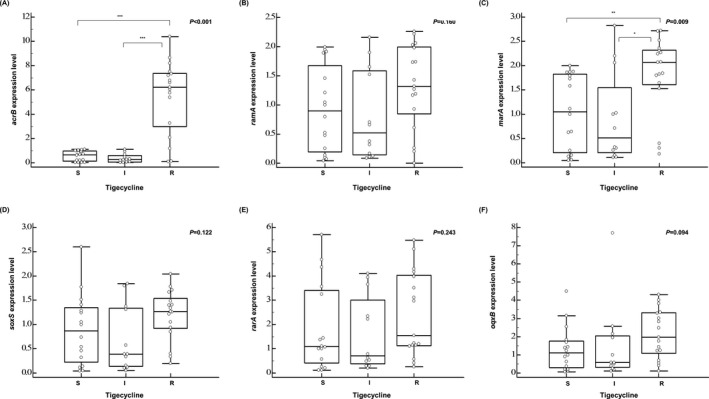
The relative expression level of each gene in the tigecycline‐susceptible (N = 16), tigecycline‐intermediate (N = 12), and tigecycline‐resistant (N = 17) groups. *P < .05; **P < .01; ***P < .001; Kruskal‐Wallis test followed by Dunn's multiple comparison test. I, intermediate; R, resistant; S, susceptible

**Figure 3:**
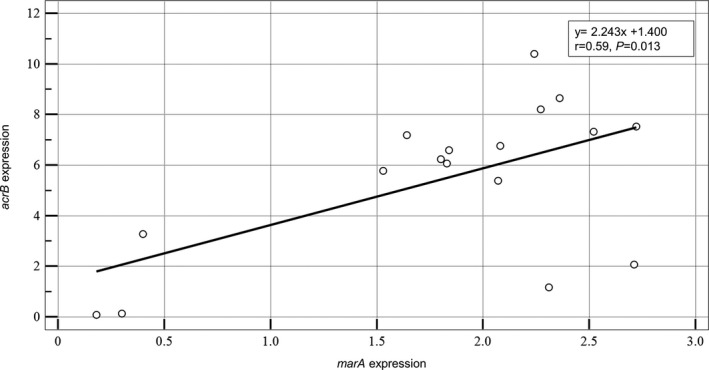
Correlation between expression of the transcriptional activator‐encoding *marA* gene and efflux pump‐encoding *acrB* gene in tigecycline‐ and carbapenem‐resistant *Klebsiella pneumoniae*. *P* value; Mann‐Whitney rank sum test

The expression levels of the efflux pump‐encoding *acrB* gene (*P* < .001) and its transcriptional activator‐encoding *marA* gene (*P* = .009) in TCRKP isolates were significantly higher than those in both the tigecycline‐intermediate and tigecycline‐susceptible groups (Figure 2). Moreover, correlation analysis between the expression of *acrB* and *marA* in the TCRKP isolates indicated a moderate correlation (*r* = 0.59, *P* = .013; Figure 3). There was no significant difference in the expression levels of *ramA*, *soxS*, *oqxB*, and *rarA* genes among the three groups.

### 3.5 Mutation analysis of *acrR*, *oqxR*, *ramR*, *rpsJ*, *tet(A)*, and *tet(X)* genes and *pI* and *pII* promoter regions, and their relationship with gene expression levels

The relative expression levels of *acrB*, *ramA*, *marA*, *soxS*, *rarA*, and *oqxB* genes and mutation analysis of TCRKP isolates are presented in Table 1. The *acrR*, *oqxR*, *ramR*, and *rpsJ* genes and *pI* and *pII* promoter regions were detected in all 17 TCRKP isolates. The *tet(A)* gene was detected in 52.9% (9/17) of the TCRKP isolates. In contrast, the *tet(X)* gene was not detected in any of the TCRKP isolates.

**Table 1:** Mutation analysis of *acrR*, *ramR*,*oqxR*, and *tet(A)*genes and *pI* and *pII* promoter regions and the relative expression levels of *acrB*, *ramA*, *marA*, *soxS*, *rarA*, and *oqxB* in TCRKP isolates

TGC MIC (mg/L)	Isolates	Mutation analysis^a^						Relative expression level^b^					
		*acrR*	*pI promoter*	*pII promoter*	*ramR*	*oqxR*	*tet(A)*	*acrB*	*ramA*	*marA*	*soxS*	*rarA*	*oqxB*
4	CRKP14	△396‐415, g441a	a54g	919_920insa, a1106g, c1133t, t1139a, △1316	None	t389c (V130A)	‐	0.13 ± 0.02	0.20 ± 0.01	0.30 ± 0.02	0.34 ± 0.02	0.58 ± 0.03	0.56 ± 0.14
	CRKP33	None	a54g	919_920insa, △1316	c56t (A19V)*, △343‐351*	None	‐	1.17 ± 0.37	**2.22 ± 0.74**	**2.31 ± 0.35**	1.71 ± 0.31	**5.11 ± 0.87**	**3.82 ± 0.72**
	CRKP35	None	a54g	919_920insa, g929a*, c992t, c999t*, a1106g, △1316	△332‐342*	None	‐	**7.33 ± 0.67**	1.73 ± 0.17	**2.52 ± 0.36**	1.41 ± 0.20	**4.29 ± 1.46**	**4.00 ± 0.49**
	CRKP55	None	a54g, g133a*	919_920insa, a1106g, c1133t, t1139a, △1316	None	t389c (V130A)	‐	**8.21 ± 0.82**	0.00 ± 0.00	**2.27 ± 0.24**	**2.04 ± 0.48**	**3.99 ± 0.25**	**3.35 ± 0.30**
	CRKP74	None	a54g	919_920insa, g929a*, c992t, c999t*, a1106g, △1316	None	None	△1928, 1936_1937insc	**6.60 ± 0.54**	1.19 ± 0.05	1.84 ± 0.31	0.85 ± 0.13	1.23 ± 0.17	1.43 ± 0.20
	CRKP79	None	a54g	919_920insa, g929a*, c992t, c999t*, a1106g, △1316	t563a (L188Q)*	None	‐	**7.19 ± 0.68**	0.93 ± 0.16	1.64 ± 0.08	1.22 ± 0.05	1.14 ± 0.09	1.26 ± 0.18
	CRKP82	None	a54g	919_920insa, g929a*, c992t, c999t*, a1106g, △1316	t563a (L188Q)*	None	△1928 1936_1937insc	**6.06 ± 0.52**	1.32 ± 0.36	1.83 ± 0.18	1.27 ± 0.04	1.54 ± 0.12	1.76 ± 0.05
	CRKP90	None	a54g	919_920insa, g929a*, c992t, c999t*, a1106g, △1316	t563a (L188Q)*	None	‐	**3.28 ± 0.48**	0.61 ± 0.09	0.40 ± 0.07	0.40 ± 0.10	0.41 ± 0.26	0.70 ± 0.20
8	CRKP2	None	a54g	919_920insa, △1316	△44‐46*	t92a (A30Q)*	△1928 1936_1937insc	0.09 ± 0.05	0.26 ± 0.06	0.18 ± 0.04	0.20 ± 0.08	0.26 ± 0.07	0.42 ± 0.20
	CRKP30	None	a54g	919_920insa, g929a*, c992t, c999t*, a1106g, △1316	△103*	None	‐	**10.41 ± 3.45**	**2.26 ± 0.36**	**2.24 ± 0.33**	1.34 ± 0.22	**2.97 ± 1.31**	**4.32 ± 0.75**
	CRKP32	None	a54g, △163*	919_920insa, g929a*, c992t, c999t*, a1106g, △1316	None	None	△1928 1936_1937insc	**7.53 ± 0.30**	1.98 ± 0.15	**2.72 ± 0.06**	1.49 ± 0.21	**3.51 ± 0.21**	**3.29 ± 0.16**
	CRKP34	None	a54g	919_920insa, g929a*, c992t, c999t*, a1106g, △1316	△332*	None	△1928 1936_1937insc	**6.77 ± 0.86**	**2.06 ± 0.22**	**2.08 ± 0.19**	1.78 ± 0.45	**4.18 ± 0.34**	**2.84 ± 0.34**
	CRKP36	None	a54g	919_920insa, g929a*, c992t, c999t*, a1106g, △1316	c364t (Q122stop)	None	△1928 1936_1937insc	**8.65 ± 0.72**	**2.03 ± 0.24**	**2.36 ± 0.30**	1.67 ± 0.13	**5.48 ± 0.48**	**3.02 ± 0.24**
	CRKP75	None	a54g	919_920insa, c992t, a1106g, △1316	△332‐344*, g474a*	t389c (V130A)	‐	**2.07 ± 0.86**	1.27 ± 0.12	**2.71 ± 0.35**	1.26 ± 0.09	**3.12 ± 0.29**	0.11 ± 0.02
	CRKP80	None	a54g	g929a*, c992t, c999t*, a1106g, △1316	t563a (L188Q)*	None	△1928 1936_1937insc	**5.39 ± 0.23**	1.43 ± 0.13	**2.07 ± 0.22**	0.94 ± 0.06	1.20 ± 0.25	1.23 ± 0.23
	CRKP86	None	a54g	g929a*, c992t, c999t*, a1106g, △1316	g554t (W185L)*, t563a (L188Q)*	None	△1928 1936_1937insc	**5.78 ± 0.48**	1.17 ± 0.05	1.53 ± 0.21	1.40 ± 0.011	1.15 ± 0.18	1.96 ± 0.13
16	CRKP73	△385‐395*	a54g	919_920insa, g929a*, c992t, c999t*, a1106g, △1316	None	None	△1928 1936_1937insc	**6.23 ± 0.71**	1.74 ± 0.26	1.80 ± 0.24	1.05 ± 0.12	1.12 ± 0.13	**2.47 ± 0.42**

Note

The numbers in bold indicate at least 2‐fold higher transcriptional level of the gene compared to that in the tigecycline‐susceptible control CRKP87 isolate containing the wild‐type gene.

Abbreviations: ‐, the gene was not confirmed in PCR; △, deletion; ins, insertion; MIC, minimum inhibitory concentration; None, the gene was confirmed in PCR, but no mutation was detected; TCRKP, tigecycline‐resistant and carbapenem‐resistant *Klebsiella pneumoniae*; TGC, tigecycline.

^a^ In mutation analysis, uppercase letters indicate amino acids and lowercase letters indicate the nucleotide bases.

^b^ Data are expressed as the mean ± standard deviation (SD).

* Mutations observed only in tigecycline‐resistant CRKP isolates.

Mutations in the efflux pump‐encoding *acrR* and *oqxR* genes were identified in 11.8% (2/17) and 23.5% (4/17) of the TCRKP isolates, respectively. Of isolates harboring the mutations only identified in TCRKP isolates, except for mutations identified in tigecycline‐susceptible and/or tigecycline‐intermediate CRKP isolates, CRKP 73, harboring the deletion 385‐395gcccagcggca in *acrR*, showed increased expression levels of *acrB* (6.23 ± 0.71). There was no increase in the expression level of the *oqxB* gene (0.42 ± 0.20) in the CRKP2 isolate harboring the point mutation t92a (A30Q) in the *oqxR* gene. Mutants of RamR, the repressor of *ramA*, were detected in 12 TCRKP isolates (12/17, 70.6%). Some TCRKP isolates (CRKP30, CRKP33, and CRKP34) harboring frameshift mutations in *ramR* tended to express slightly higher transcriptional levels of *ramA* than the tigecycline‐susceptible control CRKP87 isolate. The expression level of *ramA* was not sufficiently increased by mutations such as the deletion 163g or point mutation g133a in the *pI* promoter region and point mutations g929a and c999t in the *pII* promoter region. All nine TCRKP isolates harboring the *tet(A)* gene had the same mutation, deletion 1928a, and insertion 1936_1937c, changing the 201‐203rd amino acids in the sequence from serine, phenylalanine, and valine (SFV) to alanine, serine, and proline (ASP). However, this mutation was also detected in tigecycline‐susceptible CRKP isolates (Table S3). There were no mutations in the *rpsJ* gene.

## 4 DISCUSSION

In the present study, we analyzed phenotypic characteristics including tigecycline susceptibility, molecular epidemiology, and mechanisms of tigecycline resistance in CRKP isolates from a tertiary care hospital in South Korea. According to a multicenter study in the United States, the resistance rate of tigecycline in CRKP isolates was reported to be 18.0%.^36^ However, in this study, tigecycline non‐susceptible CRKP and TCRKP accounted for 64.4% and 37.8% of strains, respectively, with a higher tigecycline resistance rate in CRKP isolates when analyzing tigecycline MIC results according to the EUCAST criteria.^36^

Some studies have reported that treatment with tigecycline could lead to the development of tigecycline resistance.^11^, ^37^ However, in this study, all TCRKP isolates were collected from patients who had not been exposed to tigecycline treatment previously. These findings revealed that tigecycline resistance might occur even without exposure to this antibiotic. According to another study, exposure to other antibiotics that are effluxed by non‐specific pumps, such as AcrAB, could indirectly contribute to reduced tigecycline susceptibility.^38^

The MLST and PFGE analyses revealed that *K pneumoniae* ST307, which was divided into five clonal groups based on PFGE, was the predominant clone among the TCRKP isolates in this study. Moreover, some of the *K pneumonia* ST307 isolates in this study harbored *bla*_KPC‐2_. *K pneumoniae* ST307 has been internationally reported as a high‐risk pathogen associated with high resistance to fluoroquinolones, third generation cephalosporins, and carbapenem.^39^, ^40^ Moreover, *K pneumoniae* ST307 is one of the dominant clonal types, along with ST11, ST768, ST15, ST23, and ST48, among the tigecycline‐resistant *K pneumoniae* isolates in South Korea.^9^ In this study, we confirmed that high‐risk TCRKP isolates such as *K pneumoniae* ST307 had already emerged and are disseminating in this area. Therefore, we should thoroughly monitor these high‐risk pathogens to prevent their transmission.

Regarding the mechanisms of tigecycline resistance, we found that tigecycline resistance in most of the CRKP isolates was associated with increased expression of the efflux pump‐encoding *acrB* gene. The upregulation of *acrB* could be mediated by a local repressor AcrR and/or transcriptional activators such as MarA, RamA, and SoxS.^38^, ^39^ Among them, we found that increased *acrB* expression correlated with overexpression of the transcriptional activator *marA* in the TCRKP isolates. However, the overexpression of *acrB* was not detected in the three TCRKP isolates. These findings suggest that tigecycline resistance in these isolates might be due to an alternative pathway or efflux pumps other than AcrAB or OqxAB.

In previous studies, it has been reported that mutations in the *ramR* gene could contribute to *ramA* overexpression and subsequent *acrAB* upregulation.^13^, ^18^, ^21^, ^22^ However, the expression levels of *ramA* in TCRKP isolates harboring mutations in *ramR* were not significantly increased compared to those in the control strain with a wild‐type *ramR* gene in the present study. Moreover, based on mutation analysis of the transcriptional start sites (*pI* and *pII* promoter regions) of the *ramA* gene, there were no specific mutations that could affect the expression level of *ramA*. This implied that *ramA* overexpression might not be required to upregulate *acrB* and to confer tigecycline resistance. Among mutations, a Q122stop mutant in RamR^13^ and V130A mutant of OqxR^22^ were reported to confer resistance to tigecycline in previous studies; however, in this study, these mutants were also observed in the tigecycline‐susceptible CRKP isolate indicating that it has little impact on tigecycline resistance.

There is one limitation to this study. Our study suggested that the upregulation of *acrB* mediated by the transcriptional activator MarA plays an important role in the mechanisms of tigecycline resistance. However, the expression of *marA* could also be regulated by a transcriptional regulator such as MarR. Therefore, further studies on additional regulators such as MarR, SoxR, and Lon protease, which affect the expression of *marA*, *soxS*, and *ramA* genes, respectively, and subsequent acrAB expression will be needed to assess the possible role in tigecycline resistance. In conclusion, although the mechanisms of tigecycline resistance are complex and have not been fully understood, our study indicates that the main mechanisms of tigecycline resistance in the CRKP isolates can be attributed to transcriptional activator MarA‐mediated overexpression of AcrAB efflux pump.

## Supporting information

Supplementary Material
