# Supplementary material for: Molecular epidemiology and mechanisms of tigecycline resistance in carbapenem‐resistant Klebsiella pneumoniae isolates
Source: J Clin Lab Anal. 2020 Aug 20;34(12):e23506. doi: 10.1002/jcla.23506 (PMC7755817; doi:10.1002/jcla.23506)
Supplement: Supplementary file 1 — Supplementary Material [file JCLA-34-e23506-s001.docx]

Supplement table S1. The primers used to detect the resistance genes

|  | Gene | Forward sequence (5′→3′) | Reverse sequence (5′→3′) | Reference |
| --- | --- | --- | --- | --- |
| Carbapenem | bla_NDM_ | GGTTTGGCGATCTGGTTTTC | CGGAATGGCTCATCACGATC | [[1](#_ENREF_1)] |
|  | bla_IMP_ | GGAATAGAGTGGCTTAAYTC | GGTTTAAYAAAACAACCACC |  |
|  | bla_VIM_ | GATGGTGTTTGGTCGCATA | CGAATGCGCAGCACCAG |  |
|  | bla_KPC_ | CGTCTAGTTCTGCTGTCTTG | CTTGTCATCCTTGTTAGGCG |  |
|  | bla_OXA-48-like_ | GCGTGGTTAAGGATGAACAC | CATCAAGTTCAACCCAACCG |  |
|  | bla_GES_ | GCTTCATTCACGCACTATT | CGATGCTAGAAACCGCTC | [[2](#_ENREF_2)] |
| β-lactamase | bla_CTX-M-1_ | AGTTCACGCTGATGGCGACG | AACCCAGGAAGCAGGCAGTCC | [[4](#_ENREF_4)] |
|  | bla_CTX-M-9_ | GATTGACCGTATTGGGAGTTT | CGGCTGGGTAAAATAGGTCA | [[5](#_ENREF_5)] |
|  | bla_TEM_ | ATAAAATTCTTGAAGACGAA | GACAGTTACCAATGCTTAAT | [[6](#_ENREF_6)] |
|  | bla_SHV_ | GGGTTATTCTTATTTGTCGC | TTAGCGTTGCCAGTGCTC |  |
| AmpC | bla_cit_ | TGGCCAGAACTGACAGGCAAA | TTTCTCCTGAACGTG GCTGGC | [[7](#_ENREF_7)] |
|  | bla_MOX_ | GCTGCTCAAGGAGCACAGGAT | CACATTGACATAGGTGTGGTGC |  |
|  | bla_DHA_ | AACTTTCACAGGTGTGCTGGGT | CCGTACGCATACTGGCTTTGC |  |
|  | bla_ACC_ | AACAGCCTCAGCAGCCGGTTA | TTCGCCGCAATCATCCCTAGC |  |
|  | bla_EBC_ | TCGGTAAAGCCGATGTTGCGG | CTTCCACTGCGGCTGCCAGTT |  |
|  | bla_FOX_ | AACATGGGGTATCAGGGAGATG | CAAAGCGCGTAACCGGATTGG |  |
| Quinolone | qnrA | AGAGGATTTCTCACGCCAGG | TGCCAGGCACAGATCTTGAC | [[8](#_ENREF_8)] |
|  | qnrB | GGMATHGAAATTCGCCACTG* | TTTGCYGYYCGCCAGTCGAA* |  |
|  | qnrS | GCAAGTTCATTGAACAGGGT | TCTAAACCGTCGAGTTCGGCG |  |
|  | *aac(6’)-Ib-cr* | TGACCAACAGCAACGATTCC | TTAGGCATCACTGCGTGTTC | [[9](#_ENREF_9)] |
|  | qepA | GGACATCTACGGCTTCTTCG | AGCTGCAGGTACTGCGTCAT | [[10](#_ENREF_10)] |
| 16S rRNA methylase | armA | AGGTTGTTTCCATTTCTGAG | TCTCTTCCATTCCCTTCTCC | [[11](#_ENREF_11)] |
|  | rmtA | CTAGCGTCCATCCTTTCCTC | TTTGCTTCCATGCCCTTGCC |  |
|  | rmtB | CCCAAACAGACCGTAGAGGC | CTCAAACTCGGCGGGCAAGC |  |
|  | rmtC | CGAAGAAGTAACAGCCAAAG | ATCCCAACATCTCTCCCACT |  |

*M=A or C; H=A or C or T; Y= C or T

Supplement table S2. The primers used for detection and mutation analysis in the *acrR*, *oqxR*, *ramR*, *rpsJ*, *tet(A)*, and *tet(X)* genes and *pI* and *pII* promoter regions, and quantitative real-time PCR (qRT-PCR)

| Purpose | Gene | Forward sequence (5′→3′) | Reverse sequence (5′→3′) | Reference |
| --- | --- | --- | --- | --- |
| Mutation detection | *ramR* | CACGGTTCATATCCTGACCA | CCRTCGACCTTAAACACGTC | [[12](#_ENREF_12)] |
|  | *acrR* | GCTAAGCTGCCTGAGAGCAT | ATGCAAATGCCGGAGAATAC |  |
|  | *rpsJ* | ACAGCCGGTTCGATATGA | AGTAACGCGGTTTGCTTC |  |
|  | *oqxR* | GTCACCAGAAAATGATTAATGCGC | GCCTTTGCCCGTGAAATCAG | [[13](#_ENREF_13)] |
|  | *tet(A)* | GCCTTTCCTTTGGGTTCTCT | TGTCCGACAAGTTGCATGAT |  |
|  | *tet(X)* | CAATAATTGGTGGTGGACCC | TTCTTACCTTGGACATCCCG |  |
|  | *pI* | CTACTTTTTTCCTCACGCAG | CCCTGCGGCGCCTTACCA | [[14](#_ENREF_14)] |
|  | *pII* | GGGCCAGTTTTCTGTT | ATAGTATCAATCACCTGAGC |  |
| qRT-PCR | ramA | GATATCGCTCGCCATGC | CTGTGGTTCTCTTTGCGGTAG | [[12](#_ENREF_12)] |
|  | acrB | AAACTTCGCCACTACGTCATA | AGCTTAACGCCTCGATCAT |  |
|  | marA | TGTCTGAGCGCTCCGGTTACT | TTCTGCGCAATCTCCGTCA |  |
|  | soxS | TACCTGCAGCGGATGTTC | AAGGTTTGCTGCGAGACGTAG |  |
|  | rrsE | GTCATCATGGCCCTTACGAG | ACTTTATGAGGTCCGCTTGCT |  |
|  | *rarA* | GTTTGTTGACGAAGTGCA | GCCATCATTTCCAGGGTA | [[15](#_ENREF_15)] |
|  | oqxB | CGAAGAAAGACCTCCCTACCC | CGCCGCCAATGAGATACA |  |

p, promoter; qRT-PCR, quantitative real-time PCR

Supplemental Table S3. Mutation analysis of *acrR*, *ramR*, *oqxR*, and *tet(A)*genes and *pI* and *pII* promoter regions and the relative expression levels of *acrB*, *ramA*, *marA*, *soxS*, *rarA*, and *oqxB* in tigecycline-susceptible and intermediate CRKP isolates

| TGC MIC  (mg/L) | Isolates | Mutation analysis^a^ | | | | | | Relative expression level^b^ | | | | | |
| --- | --- | --- | --- | --- | --- | --- | --- | --- | --- | --- | --- | --- | --- |
|  |  | *acrR* | *pI promoter* | *pII promoter* | *ramR* | *oqxR* | *tet(A)* | *acrB* | *ramA* | *marA* | *soxS* | *rarA* | *oqxB* |
| 0.5 | CRKP87 | None | a54g | 919_920insa, a1106g, c1133t, t1139a, △1316 | None | t389c (V130A) | - | 1.00 | 1.00 | 1.00 | 1.00 | 1.00 | 1.00 |
|  | CRKP19 | None | a54g | 919_920insa, a1106g, c1133t, t1139a, △1316 | △481 | t389c (V130A) | - | 0.05 ± 0.00 | 0.10 ± 0.01 | 0.13 ± 0.01 | 0.11 ± 0.01 | 0.20 ± 0.04 | 0.21 ± 0.03 |
|  | CRKP24 | None | a54g | 919_920insa, a1106g, c1133t, t1139a, △1316 | △242 | None | - | 0.02 ± 0.00 | 0.07 ± 0.01 | 0.05 ± 0.00 | 0.05 ± 0.00 | 0.13 ± 0.03 | 0.11 ± 0.01 |
|  | CRKP31 | None | a54g | 919_920insa, a1106g, c1133t, t1139a, △1316 | None | t389c (V130A) | - | 1.07 ± 0.10 | 1.89 ± 0.15 | 1.86 ± 0.34 | 1.77 ± 0.10 | 4.36 ± 0.66 | 1.83 ± 0.04 |
|  | CRKP59 | None | a54g | 919_920insa, c937g, △1316 | None | t389c (V130A) | - | 0.83 ± 0.18 | 1.95 ± 0.29 | 2.00 ± 0.31 | 2.60 ± 0.70 | 4.67 ± 1.05 | 4.50 ± 1.75 |
| 1 | CRKP11 | None | a54g | 919_920insa, a1106g, c1133t, t1139a, △1316 | None | t389c (V130A) | - | 0.03 ± 0.01 | 0.04 ± 0.00 | 0.06 ± 0.01 | 0.04 ± 0.00 | 0.13 ± 0.01 | 0.08 ± 0.01 |
|  | CRKP20 | None | a54g | 919_920insa, a1106g, △1316 | None | t389c (V130A) | - | 0.20 ± 0.02 | 0.25 ± 0.03 | 0.25 ± 0.06 | 0.32 ± 0.02 | 0.57 ± 0.07 | 0.37 ± 0.04 |
|  | CRKP22 | None | a54g | 919_920insa, △1316 | None | t389c (V130A) | - | 0.24 ± 0.03 | 0.52 ± 0.05 | 0.64 ± 0.05 | 0.54 ± 0.05 | 1.11 ± 0.08 | 1.69 ± 0.09 |
|  | CRKP25 | None | a54g | 919_920insa, c937g, △1316 | c364t (Q122stop) | t389c (V130A) | - | 0.22 ± 0.17 | 0.48 ± 0.08 | 0.63 ± 0.12 | 0.46 ± 0.05 | 1.01 ± 0.21 | 0.62 ± 0.07 |
|  | CRKP29 | None | a54g, 95_96insIS481 ^c^ | 919_920insa, a1106g, △1316 | c364t (Q122stop) | t389c (V130A) | - | 0.05 ± 0.01 | 0.14 ± 0.02 | 0.16 ± 0.02 | 0.14 ± 0.02 | 0.26 ± 0.05 | 0.22 ± 0.03 |
|  | CRKP37 | △396-415, g441a | a54g | 919_920insa, a1106g, c1133t, t1139a, △1316 | None | t389c (V130A) | - | 0.72 ± 0.25 | 1.91 ± 0.45 | 1.87 ± 0.51 | 1.41 ± 0.37 | 3.56 ± 0.76 | 1.38 ± 0.32 |
|  | CRKP38 | None | a54g | 919_920insa, c992t, a1106g, △1316 | None | t389c (V130A) | △1928,  1936_1937insc | 1.10 ± 0.29 | 1.99 ± 0.20 | 1.79 ± 0.14 | 1.51 ± 0.16 | 5.71 ± 0.82 | 3.15 ± 0.61 |
|  | CRKP65 | None | a54g, 95_96insIS481 ^c^ | 919_920insa, a1106g, △1316 | c364t (Q122stop) | t389c (V130A) | - | 0.63 ± 0.12 | 1.46 ± 0.21 | 1.88 ± 0.28 | 1.05 ± 0.15 | 3.26 ± 0.50 | 2.54 ± 1.77 |
|  | CRKP84 | None | a54g | 919_920insa, c992t, a1106g, △1316 | None | None | △1928,  1936_1937insc | 1.05 ± 0.14 | 1.04 ± 0.13 | 1.73 ± 0.22 | 1.29 ± 0.25 | 1.44 ± 0.22 | 1.23 ± 0.17 |
|  | CRKP85 | None | a54g | c992t, a1106g, △1316 | None | None | △1928,  1936_1937insc | 0.93 ± 0.23 | 1.21 ± 0.11 | 1.58 ± 0.83 | 1.24 ± 0.09 | 1.38 ± 0.17 | 1.43 ± 0.10 |
|  | CRKP88 | None | a54g | a1106g, c1133t, t1139a, △1316 | None | t389c (V130A) | △1928,  1936_1937insc | 0.71 ± 0.10 | 0.80 ±0.11 | 1.11 ± 0.11 | 0.74 ± 0.06 | 1.07 ± 0.13 | 0.91 ± 0.26 |
| 2 | CRKP4 | △396-415, g441a | a54g | 919_920insa, a1106g, c1133t, t1139a, △1316 | None | t389c (V130A) | - | 0.49 ± 0.11 | 0.70 ± 0.01 | 1.00 ± 0.07 | 0.57 ± 0.05 | 2.22 ± 0.28 | 1.00 ± 0.12 |
|  | CRKP7 | None | a54g | 919_920insa, a1106g, c1133t, t1139a, △1316 | None | t389c (V130A) | - | 0.04 ± 0.01 | 0.09 ± 0.00 | 0.16 ± 0.02 | 0.05 ± 0.01 | 0.20 ± 0.04 | 0.19 ± 0.05 |
|  | CRKP8 | None | a54g | 919_920insa, c992t, a1106g, △1316 | None | c209t (P70L)  t365c (L122S) | - | 0.04 ± 0.01 | 0.11 ± 0.01 | 0.11 ± 0.01 | 0.10 ± 0.02 | 0.29 ± 0.02 | 0.25 ± 0.03 |
|  | CRKP10 | None | a54g | 919_920insa, c992t, a1106g, △1316 | None | t365c (L122S) | - | 0.07 ± 0.01 | 0.12 ± 0.01 | 0.12 ± 0.01 | 0.12 ± 0.01 | 0.29 ± 0.05 | 0.12 ± 0.01 |
|  | CRKP21 | None | a54g | 919_920insa, a1106g, △1316 | None | t389c (V130A) | - | 0.24 ± 0.02 | 0.38 ± 0.06 | 0.30 ± 0.09 | 0.39 ± 0.03 | 0.78 ± 0.07 | 0.57 ± 0.11 |
|  | CRKP23 | None | a54g, 95_96insIS481^c^ | 919_920insa, a1106g, △1316 | c364t (Q122stop) | t389c (V130A) | - | 0.10 ± 0.02 | 0.17 ± 0.01 | 0.26 ± 0.04 | 0.16 ± 0.02 | 0.48 ± 0.06 | 0.39 ± 0.01 |
|  | CRKP28 | None | a54g | 919_920insa, c992t, a1106g, △1316 | c314a (A105V) | None | △1928,  1936_1937insc | 0.21 ± 0.03 | 0.32 ± 0.04 | 0.32 ± 0.02 | 0.34 ± 0.02 | 0.63 ± 0.03 | 0.50 ± 0.03 |
|  | CRKP60 | None | a54g | 919_920insa, a1106g, c1133t, t1139a, △1316 | None | t389c (V130A) | △1928,  1936_1937insc | 0.93 ± 0.21 | 1.65 ± 0.25 | 2.06 ± 0.27 | 1.80 ± 0.23 | 3.94 ± 0.56 | 7.71 ± 1.01 |
|  | CRKP64 | None | a54g, 95_96insIS481 ^c^ | 919_920insa, a1106g, △1316 | c364t (Q122stop) | t389c (V130A) | - | 1.11 ± 0.18 | 2.16 ± 0.52 | 2.20 ± 0.14 | 1.84 ± 0.31 | 4.11 ± 0.48 | 2.15 ± 0.12 |
|  | CRKP69 | None | a54g | 919_920insa, c992t, a1106g, △1316 | gc64-65aa (A22L) | None | △1928,  1936_1937insc | 0.71 ± 0.04 | 1.90 ± 0.14 | 2.83 ± 0.67 | 1.37 ± 0.10 | 3.65 ± 0.24 | 2.58 ± 0.34 |
|  | CRKP71 | None | a54g | 919_920insa, c992t, a1106g, △1316 | gc64-65aa (A22L) | None | △1928,  1936_1937insc | 0.30 ± 0.13 | 1.52 ± 0.27 | 1.03 ± 0.17 | 1.31 ± 0.23 | 2.35 ± 0.41 | 1.95 ± 0.31 |
|  | CRKP72 | None | a54g | 919_920insa, a1106g, c1133t, t1139a, △1316 | None | None | - | 0.29 ± 0.13 | 0.66 ± 0.28 | 0.71 ± 0.19 | 0.39 ± 0.10 | 0.54 ± 0.15 | 0.59 ± 0.22 |

TGC, tigecycline; MIC, minimum inhibitory concentration; None, the gene was confirmed in PCR, but no mutation was detected; -, the gene was not confirmed in PCR; △, deletion; ins, insertion.

^a^In mutation analysis, uppercase letters indicate amino acids and lowercase letters indicate the base of the nucleotide.

^b^Data are expressed as mean ± standard deviation (SD)

^c^IS481 insertion into the nucleotide 95-96 of the *ramR* promoter I region.

References

1. Poirel L, Walsh T, Cuvillier V, Nordmann P. Multiplex PCR for detection of acquired carbapenemase genes. 2011;70:119-23.

2. Hong SS, Kim K, Huh JY, Jung B, Kang MS, Hong SG. Multiplex PCR for rapid detection of genes encoding class A carbapenemases. Annals of laboratory medicine 2012;32:359-61.

3. Shanthi M, Sekar U, K A, Bramhne HG. OXA-181 Beta Lactamase is not a Major Mediator of Carbapenem Resistance in Enterobacteriaceae. J Clin Diagn Res 2013;7:1986-8.

4. Bogaerts P, Galimand M, Bauraing C, Deplano A, Vanhoof R, De Mendonca R, et al. Emergence of ArmA and RmtB aminoglycoside resistance 16S rRNA methylases in Belgium. Journal of Antimicrobial Chemotherapy 2007;59:459-64.

5. Kim MH, Lee HJ, Park KS, Suh JT. Molecular characteristics of extended spectrum beta-lactamases in Escherichia coli and Klebsiella pneumoniae and the prevalence of qnr in Extended spectrum beta-lactamase isolates in a tertiary care hospital in Korea. Yonsei Med J 2010;51:768-74.

6. YAO F, QIAN Y, CHEN S, WANG P, HUANG Y. Incidence of Extended-Spectrum β-Lactamases and Characterization of Integrons in Extended-Spectrum β-Lactamase-producing Klebsiella pneumoniae Isolated in Shantou, China. Acta Biochimica et Biophysica Sinica 2007;39:527-32.

7. Pérez-Pérez FJ and Hanson ND. Detection of Plasmid-Mediated AmpC β-Lactamase Genes in Clinical Isolates by Using Multiplex PCR. Journal of Clinical Microbiology 2002;40:2153-62.

8. Cattoir V, Poirel L, Rotimi V, Soussy C-J, Nordmann P. Multiplex PCR for detection of plasmid-mediated quinolone resistance qnr genes in ESBL-producing enterobacterial isolates. Journal of Antimicrobial Chemotherapy 2007;60:394-7.

9. Fihman V, Lartigue MF, Jacquier H, Meunier F, Schnepf N, Raskine L, et al. Appearance of aac(6')-Ib-cr gene among extended-spectrum b-lactamase-producing Enterobacteriaceae in a French hospital. Journal of Infection 2008;56:454-9.

10. Kang HY, Tamang MD, Seol SY, Kim J. Dissemination of Plasmid-mediated qnr, aac(6')-Ib-cr, and qepA Genes Among 16S rRNA Methylase Producing Enterobacteriaceae in Korea. J Bacteriol Virol 2009;39:173-82.

11. Wang Y, Shen M, Yang J, Dai M, Chang Y, Zhang C, et al. Prevalence of carbapenemases among high-level aminoglycoside-resistant Acinetobacter baumannii isolates in a university hospital in China. Experimental and therapeutic medicine 2016;12:3642-52.

12. Wang X, Chen H, Zhang Y, Wang Q, Zhao C, Li H, et al. Genetic characterisation of clinical Klebsiella pneumoniae isolates with reduced susceptibility to tigecycline: Role of the global regulator RamA and its local repressor RamR. International Journal of Antimicrobial Agents 2015;45: 635-40.

13. Chiu S-K, Huang L-Y, Chen H, Tsai Y-K, Liou C-H, Lin J-C, et al. Roles of ramR and tet(A) Mutations in Conferring Tigecycline Resistance in Carbapenem-Resistant Klebsiella pneumoniae Clinical Isolates. Antimicrobial Agents and Chemotherapy 2017;61:e00391-17.

14. Rosenblum R, Khan E, Gonzalez G, Hasan R, Schneiders T. Genetic regulation of the ramA locus and its expression in clinical isolates of Klebsiella pneumoniae. International journal of antimicrobial agents 2011;38:39-45.

15. Chiu S-K, Chan M-C, Huang L-Y, Lin Y-T, Lin J-C, Lu P-L, et al. Tigecycline resistance among carbapenem-resistant Klebsiella Pneumoniae: Clinical characteristics and expression levels of efflux pump genes. PLOS ONE 2017;12:e0175140.
